# Sex Differences in How Social Networks and Relationship Quality Influence Experimental Pain Sensitivity

**DOI:** 10.1371/journal.pone.0078663

**Published:** 2013-11-05

**Authors:** Jacob M. Vigil, Lauren N. Rowell, Simone Chouteau, Alexandre Chavez, Elisa Jaramillo, Michael Neal, David Waid

**Affiliations:** Department of Psychology, University of New Mexico, Albuquerque, New Mexico, United States of America; University of Bologna, Italy

## Abstract

This is the first study to examine how both structural and functional components of individuals’ social networks may moderate the association between biological sex and experimental pain sensitivity. One hundred and fifty-two healthy adults (mean age = 22yrs., 53% males) were measured for cold pressor task (CPT) pain sensitivity (i.e., intensity ratings) and core aspects of social networks (e.g., proportion of friends vs. family, affection, affirmation, and aid). Results showed consistent sex differences in how social network structures and intimate relationship functioning modulated pain sensitivity. Females showed higher pain sensitivity when their social networks consisted of a higher proportion of intimate types of relationship partners (e.g., kin vs. non kin), when they had known their network partners for a longer period of time, and when they reported higher levels of logistical support from their significant other (e.g., romantic partner). Conversely, males showed distinct patterns in the opposite direction, including an association between higher levels of logistical support from one’s significant other and lower CPT pain intensity. These findings show for the first time that the direction of sex differences in exogenous pain sensitivity is likely dependent on fundamental components of the individual’s social environment. The utility of a social-signaling perspective of pain behaviors for examining, comparing, and interpreting individual and group differences in experimental and clinical pain reports is discussed.

## Introduction

Sex differences in experimental pain performance are pervasive in the pain literature, with women typically reporting higher pain sensitivity than males [1-4]. One hypothesis from a social-signaling perspective of pain behaviors is that these differences may be associated with social network characteristics and prototypical relationship functioning in males and females [5-8]. The findings on the influence of social support on pain sensitivity have been mixed. It is plausible that stronger social support is associated with less clinical pain and improved patient outcomes [9-12], but some research has shown inconclusive associations [13] or the inverse finding that stronger social support is associated with increased clinical pain [14-16]. Experimental studies have also produced mixed results concerning social contextual influences on pain perception [7,17-19]. 

 Recent social-signaling models from evolutionary psychology predict that people’s close relationships should adaptively influence the phenotypic expression of pain perception [5,6,8,20,21]. Specifically, pain experiences should correspond to how often people interact with intimate (e.g., in terms of shared time and interpersonal knowledge) relationship partners such as romantic partners, family, and close friends, because these types of partners are most likely to provide solicitous responses to the pain sufferer. From the perspective of Vigil’s “Socio-Relational Framework of Expressive Behaviors” (SRFB; [5-8]), for instance, the expression of pain behaviors is partly functional for demonstrating vulnerability and ultimately *trustworthiness cues* in ways that demand attention from, and effectively exploit the solicitous tendencies of intimate and familiar (i.e., reliable) affiliates. When interacting with less intimate and riskier affiliates, humans instead rely on demonstrations of empowerment and ultimately *capacity cues* (e.g., hypoalgesia) rather than trustworthiness cues to attract and maintain relationships. Thus, from the perspective of the SRFB, the behavioral heuristic to demonstrate heightened pain sensitivity should co-occur with the likelihood of exposure to, and frequency of interactions with intimate and familiar relationship partners. The inverse would also be true, that dampened pain sensitivity should co-occur with the frequency of interactions with less intimate and riskier affiliates [5,6,8].

 An extension of this prediction is that the conventional finding of greater pain sensitivity in women [1-4,7,22] should be modulated, in part, by structural and functional components of the individual’s social network [5,6,8]. This hypothesis is based on the evidence that males often report the tendency to form less intimate peer relationships than females; for example, people that females would typically describe as ‘acquaintances’ are often included among males’ lists of ‘friends’ [5,23-26]. Thus, from a social-signaling perspective, males’ and females’ natural tendencies to form less intimate versus more intimate peer relationships (respectively) may have resulted in the greater tendency for males to utilize empowerment gestures including pain tolerance behaviors for regulating (i.e., attracting and maintaining) their peer relationships, and for females to utilize vulnerability gestures including pain reaction behaviors for accomplishing these social goals [5,6,8,26,27]. Moreover, the composition and level of intimacy of the individual’s most significant relationships and hence structural and functional components of their actual peer network should moderate the relation between biological sex and experimental pain sensitivity. 

 This is the first study to our knowledge to examine the association between biological sex, the structure and functioning of individual’s social network, and cold pressor task (CPT) pain sensitivity. On the basis of predictions derived from the social-signaling perspective of pain behaviors and the SRFB [5,6,8], we hypothesized that the level of intimacy that individuals share with their network partners will compound biological sex differences in pain sensitivity, and thus the typical pattern of females reporting higher pain intensity than males will be the most robust for females that have a high proportion of intimate types of affiliates (e.g., lover and relatives vs. non-kin) and more established (e.g., longer formed) relationships. Likewise, on the basis of predicted sex differences in the utilization of capacity cues (in males) versus trustworthiness cues (in females) for regulating one’s interpersonal relationships [5,7,8,26,27], we hypothesized that having more extensive (e.g., supportive) relationships with one’s significant other will be associated with lower pain sensitivity among males, and conversely with higher pain sensitivity among females. This research fills a fundamental gap in understanding how naturalistic peer environments and hence a major component of the social domain of pain perception influences experimental (i.e., exogenous, phasic, skin surface) pain sensitivity differently in healthy males and females. 

## Methods

### Participants

 Participants included a convenience sample of undergraduates. Prospective participants were excluded from the study if they were taking pain medication or had any problems that would increase risk from the CPT, including illnesses related to a cardiovascular disorder (e.g., high blood pressure, heart problems, or heart rhythm concerns), history of fainting or seizures, history of frostbite, having an open cut, sore or bone fracture on the limb to be immersed in water, or a history of Reynaud’s phenomenon. The sample consisted of 152 people with complete data (see below) for inclusion in the study (18–57 years, mean age = 22yrs, *SD* = 7.3, 81 males; 47% European-American, 33% Latin-American, 20% other ethnicity). 

### Procedures

#### Ethics statement

The protocol was approved by the University of New Mexico’s Institutional Review Board and two forms of written consent were obtained from all participants. The first consent form described the general research protocol, and the second described the CPT in more detail. 

 After informed written consent was obtained, participants completed self-report questionnaires including demographic items and the Norbeck Social Support Questionnaire [28,29]. Following questionnaire completion, participants viewed an instruction video for the CPT. The video explained how to use the cold pressor apparatus and the computer software to indicate pain ratings. The surveys and instruction video took about 30 minutes to complete. 

 After participants viewed the instruction video, they were led into the cold pressor room, which included a video monitor and an intercom system, as well as the cold pressor apparatus and a laptop programmed for participants to rate their pain levels. The software recorded participants’ baseline pain and pain intensity ratings at equal intervals throughout the CPT. The cold pressor task was then carried out by the participant without an experimenter present, though these interactions were monitored by an experimenter in the next room (by video and intercom) to ensure adherence to the CPT methods. This enabled us to collect CPT data without the physical presence of investigators, which has been shown to influence experimental and clinical pain reports [30-32]. Following the CPT, individuals were debriefed.

### Questionnaires

#### Norbeck Social Support Questionnaire

This questionnaire measures the quantity and quality of individuals’ social networks [28,29]. It asks participants to list the names of up to 24 significant persons who provide personal support. For each person listed, the participant then indicates the kind of relationship (spouse or partner, family member or relatives, friend, work or school associate, neighbor, etc.). Participants then use a 5-point scale to rate the amount of support they receive from each person across 8 items: how much does the person makes them feel loved and respected, how much they confide in the person, how much the person agrees with the participant, how much the person could provide immediate functional support (e.g., borrow $10, ride to doctors), how much the person could provide extensive functional support (e.g., if confined to a bed for several weeks), the length of the relationship, and the frequency of contact. The mean values for the entire network and the values pertaining to one’s significant other (S/O) were examined in this study (46% of respondents listed a S/O). Relationships with romantic partners were examined in greater detail because they are the most intimate type of relationship that healthy young adults are likely to list. 

#### Demographic Questionnaire

This asked about sex, age, ethnicity, and level of schooling. 

#### Cold pressor task

Participants were randomly assigned to one of two experimental conditions: high pain (extremely cold water) and low pain (more tepid water). Participants were seated in a chair between the pressor apparatus (left side) and the laptop computer (right side) in a small room (2.0m x 2.5m). The apparatus consisted of a small, insulated ice cooler box (5.5" x 11" x 8") that was fitted with a water circulator and filled with ice-water that was set to induce either low or high levels of thermal discomfort. In the low pain condition, the ice-water was set to 16°C (noticeably below room temperature, but only slightly distressing), and in the high pain condition, the water was set to 5°C (quite cold, and increasingly painful with time; this produces a range of pain tolerance levels with only minimal ceiling effects [33]). The analyses only included participants (*n* = 152) who had cold water temperatures within 1°C of the target temperatures, because small differences in water temperature (e.g., 2°C) can have significant effects on pain sensitivity measures [34]. Similarly, a circulator was used to prevent the water from warming around the participant’s hand [33].

 The pain assessment program (on the laptop) displayed an initial screen with the CPT instructions. The researcher verbally reiterated the instructions by describing that when participants choose to begin the task (and initiate the pain assessment program), participants were instructed to first indicate their baseline (pre-manipulation) pain severity along a standard visual analog scale (VAS, 0–10 from *no pain* to *worst pain imaginable*; this baseline measure was denoted VAS1), while simultaneously submerging their left hand into the cold water to a marked line on the wrist (1" above the wrist joint). Participants were instructed to indicate their felt pain intensity upon an audio prompt and illumination of a pain VAS that was programmed to take place every 30s (though the participant was not aware of this timing) throughout the duration of the CPT (VAS2-VAS11). Finally, participants were instructed to lift their hand out of the cold pressor apparatus and activate a termination button on the computer screen when they decided that they could not stand the cold anymore.

 Once the participants verbally indicated their understanding of the instructions, they were fitted with a finger pulsometer to monitor their heart rate during the CPT; this was done to ensure the safety of the participants. Lastly, the researcher reminded the participant that they would be recorded, and that they could begin the task whenever they desired. The researcher then left the cold pressor room and closed the door behind herself/himself. The procedure was observed on a video monitor from the next room, and the researcher returned to the experimental room to debrief the participant once they retracted their hand from the water or after the maximum duration of 5 minutes had occurred. Following debriefing, participants were asked to rest for five minutes to ensure they no longer felt any physical discomfort from involvement in the study and that their heart rate had returned to normal. 

### Data Analyses

 Two variables were used to measure the structure of the respondent's social network. These included the absolute number of social partners listed on the social support questionnaire, and the proportion of more intimate types of affiliates out of the total number of network partners (significant other + listed family members/ total network size). Previous research on the 7 functional items (all the items except length of one’s relationships) has shown that a two factor model (described as ‘affirmation’ and ‘aid’) captured unique dimensions of social support [35]. Conceptually, the length of the individual’s relationships and hence history of shared time and interpersonal knowledge is also an important component of subjective impressions of available support. Therefore, three (functionality) composite scores were used to examine the relations between respondents’ felt pain intensity and their network mean values and the values pertaining to their significant other. The first score, emotional support, included the mean values for the following items: how much the partners make the respondent feel respected, how much the partners agree with the respondent, how much the listed network partners make the respondent feel loved, and how much they confide in the partners (α = .85). The three items that loaded on the second score, logistical support, were the mean values for: amount of contact with the partners, the extent to which the listed partners can provide immediate support, and the extent to which they can provide extensive support (α = .68). The third score, length of relationship, was comprised of the single item that pertained to this construct. 

 Since CPT pain sensations tend to graduate quickly in some people, while other people hit a ceiling effect (e.g., numbing) two-thirds of the way into the task, we computed a pain intensity score that captured the VAS ratings midway into the task. This was done by averaging the pain intensity rating between 90sec. and 150sec. into the pain task (VAS4-VAS6) for all the subjects (*n* = 152) who endured the CPT for at least three minutes (72% of participants were in the mild pain condition, 28% were in the high pain condition). Analyses of Covariances (ANCOVAs) were used to examine the relations between the social network variables, the participants’ pain intensity scores in the low pain and high pain conditions, and the potential moderating role of respondent's sex on these relations. The gender of laboratory personnel has been shown to influence experimental pain reports, even when the personnel are not physically present during the discomfort task itself [Vigil, Rowell, Alcock, Maestes, unpublished data], so it is important to control for this potential confound. Fourteen researchers interacted with the participants (50% male researchers), and the examiner’s gender (male coded 0, female coded 1) and duration of time the participant kept their hand in the water were entered as covariates. Effect sizes pertaining to group comparisons were estimated with Cohen’s *d* (mean difference / mean standard deviation [36]). Because partial correlations, controlling for CPT temperature and examiner’s gender found that participants’ age was not related to the pain score (*p* > .10), this construct was not included in the analyses. A Bonferroni correction (*p* < .01) was used to indicate statistical significance to account for multiple comparisons pertaining to the five main social network variables (2 structure and 3 functionality scores). The statistical package used was IBM SPSS statistics version 21 [37].

## Results

### Descriptive Statistics

 Independent samples *t*-tests showed that the mean pain intensity scores in the high discomfort condition were nearly twice the magnitudes of values in the low discomfort condition (Ms = 5.9, 3.3; SDs = 1.9, 2.1, d = 1.33). The average number of listed social network members was 13, *SD* = 5.5, and the average proportion of intimate types of affiliates was .51 (51% intimate affiliates), *SD* = .20. The three mean functionality scores (Emotional Support, Logistic Support, and Length of Relationships) were not uniformly distributed around the average values (Medians = 3.25, 3.34, 4.38, Ranges = 2.53, 2.83, 2.73; skewness = -.79, -.51, -.89, SEs = .16; kurtosis = -.85, -.24, 1.22, SEs = .31). As would be expected, the total number of network partners was inversely related to the proportion of intimate affiliates (*r* = -.29, *p* < .001) and mean level of logistical support (*r* = -.18, *p* = .033); however, number of partners was not related to mean emotional support or average length of the participant’s relationships (*p*s > .10).

### General Sex Differences

An ANCOVA was performed to examine sex differences in the pain sensitivity score with examiner gender and duration of hand immersion as covariates. This analysis did not show a sex difference in the pain score (*p* > .10). As shown in Table 1, independent-samples analyses indicated that there were also no sex differences in any of the social network items pertaining to structure (i.e., total number of network members and proportion of intimate types of relationship partners) or to the mean functionality scores (i.e., emotional support, logistical support, and length of relationships) and functionality pertaining to one’s S/O (*p*s > .05). 

**Table 1:** Social Network Characteristics for Males and Females.

	Males	Females	
Social Variables	*M*(SD)	*M*(SD)	*t*-value
Total Number of Network Partners	13.0(5.6)	13.7(5.3)	-.83(NS)
Proportion of Intimate Affiliates	.50(.21)	.52(.19)	-.57(NS)
Mean Emotional Support	3.15(.53)	3.27(.42)	-1.43(NS)
Mean Logistical Support	3.26(.59)	3.25(.53)	.11(NS)
Mean Length of Relationships	4.35(.47)	4.32(.50)	.33(NS)
S/O Emotional Support	3.63(.50)	3.65(.50)	-.24(NS)
S/O Logistical Support	4.04(.38)	4.14(.39)	-1.01(NS)
S/O Length of Relationship	3.47(1.36)	3.54(1.50)	-.21(NS)

Note. Mean values are shown (standard deviations are in parentheses).

### Structure of Social Networks

 We first examined whether total number of network partners (Network Size) moderated the relationship between biological sex and pain sensitivity. An ANCOVA entering Network Size (dichotomously coded as values less than or greater than the mean value), Sex, Experimental Condition (low, high pain), and each of the two-way interaction terms (Network Size x Sex, Network Size x Condition, Sex x Condition), and the Network Size x Sex x Condition three-way interaction term were entered as predictor variables, and duration of hand emersion and examiner gender were entered as covariates for the pain intensity score. None of the interaction terms were significant, nor was the main effect term for Network Size (*p*s > .10), indicating that the absolute number of significant affiliates that individuals interact on the regular basis is not correlated with experimental pain intensity.

**Figure 1:**
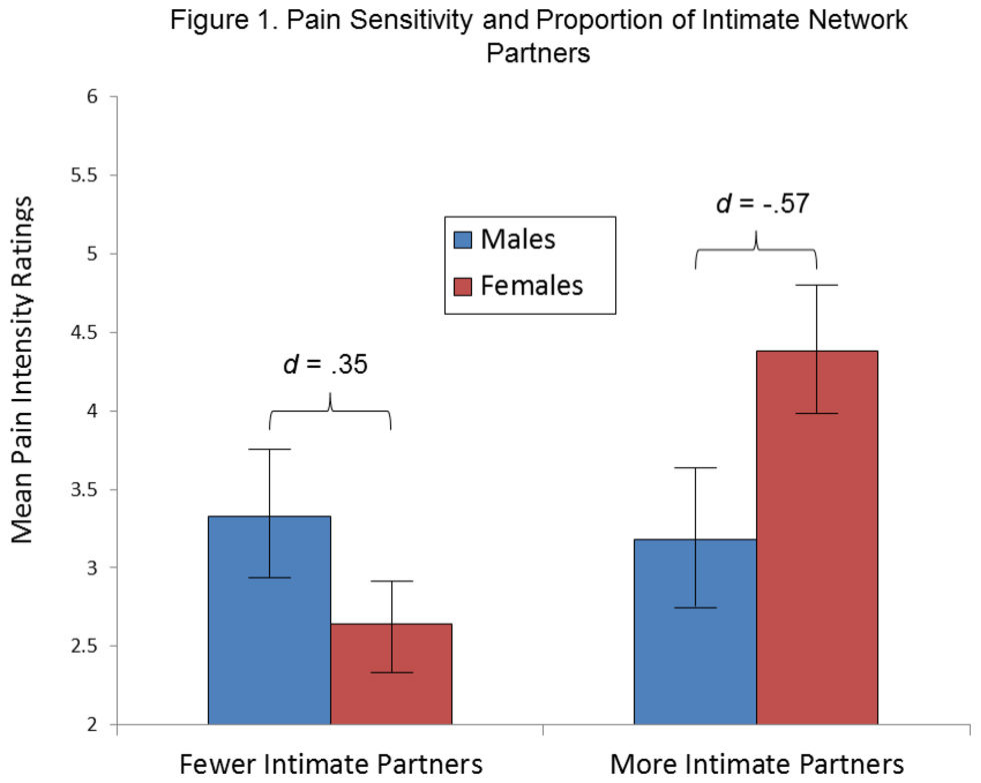
Mean pain intensity ratings in the low pain condition according to Sex and Proportion of Intimate Affiliates. The x-axis represents the proportion of network partners who were classified as a significant other or a family member, and is coded by values lower than and greater than 50% of the total number of network partners. Bars indicate standard errors of the mean.

As shown in Table 2, a similar ANCOVA pertaining to the *proportion* of intimate types of affiliates ([family + S/O]/total network; dichotomously coded as lower than or higher than 50% of total number of affiliates) revealed a trend for a significant Proportion of Intimate Affiliates x Experimental Condition interaction term, *F*(1,131) = 2.96, *p* = .09. Separate ANCOVAS entering Proportion of Intimate Affiliates, Sex, and the Proportion x Sex interaction terms as predictor variables (and hand immersion time and examiner gender as covariates) for participants in both the low pain and high pain conditions did not reveal a significant interaction term or main effect term for Proportion of Intimate Affiliates in the high pain condition. As shown in Table 3, there were trends for a significant interaction term and a main effect term for Proportion of Intimate Affiliates in the low pain condition. Figure 1 shows that the interaction was due to a significant relation between having a higher proportion of more intimate types of affiliates and higher pain intensity ratings for females, but not for males. Also shown in Figure 1, the nature of the direction of the sex differences in pain intensity was dependent on the social variable, and magnitudes of the effect sizes of the differences (in opposite directions) were moderate.

**Table 2:** ANCOVA Results for Mean Proportion of Intimate Affiliates.

Dependent Variable: Pain Intensity					
Source	Type III Sum of Squares	df	Mean Square	F	Sig.
Corrected Model	233.986^a^	9	25.998	6.440	.000
Intercept	365.471	1	365.471	90.536	.000
Duration of Hand Immersion	8.966	1	8.966	2.221	.139
Examiner Gender	2.151	1	2.151	.533	.467
Sex	.797	1	.797	.198	.657
Experimental Condition	112.787	1	112.787	27.940	.000
Proportion of Intimate Affiliates	.243	1	.243	.060	.807
Sex * Condition	.056	1	.056	.014	.907
Sex * Proportion	5.368	1	5.368	1.330	.251
Condition * Proportion	11.962	1	11.962	2.963	.088
Sex * Condition * Proportion	4.158	1	4.158	1.030	.312
Error	528.814	131	4.037		
Total	3088.667	141			
Corrected Total	762.801	140			

a R Squared = .307 (Adjusted R Squared = .259)

**Table 3:** ANCOVA Results for Proportion of Intimate Affiliates in the Low Pain Condition.

Dependent Variable: Pain Intensity	
Source	Type III Sum of Squares	df	Mean Square	F	Sig.
Corrected Model	45.232^a^	5	9.046	2.156	.065
Intercept	165.886	1	165.886	39.539	.000
Duration of Hand Immersion	6.187	1	6.187	1.475	.228
Examiner Gender	1.027	1	1.027	.245	.622
Sex	1.017	1	1.017	.242	.624
Proportion of intimate Affiliates	16.410	1	16.410	3.911	.051
Sex * Proportion	19.925	1	19.925	4.749	.032
Error	411.162	98	4.196		
Total	1647.778	104			
Corrected Total	456.393	103			

a R Squared = .099 (Adjusted R Squared = .053)

### Functionality of Social Network

 The next set of equations examined the relations between the network means for each of the scores pertaining to functionality of social support (emotional and logistic support, and length of relationships) and the pain measurements, and whether sex moderated these associations. The scores (dichotomously coded as lower than and higher than the sample mean values) were entered into three separate ANCOVAs as predictor variables, along with Sex, Experimental Condition, and their respective two-way and three-way interaction terms; hand immersion times and examiner gender were again entered as covariates. None of the interaction terms or main effects pertaining to mean Emotional Support or Logistic Support were significant (*p*s > .10). 

**Figure 2:**
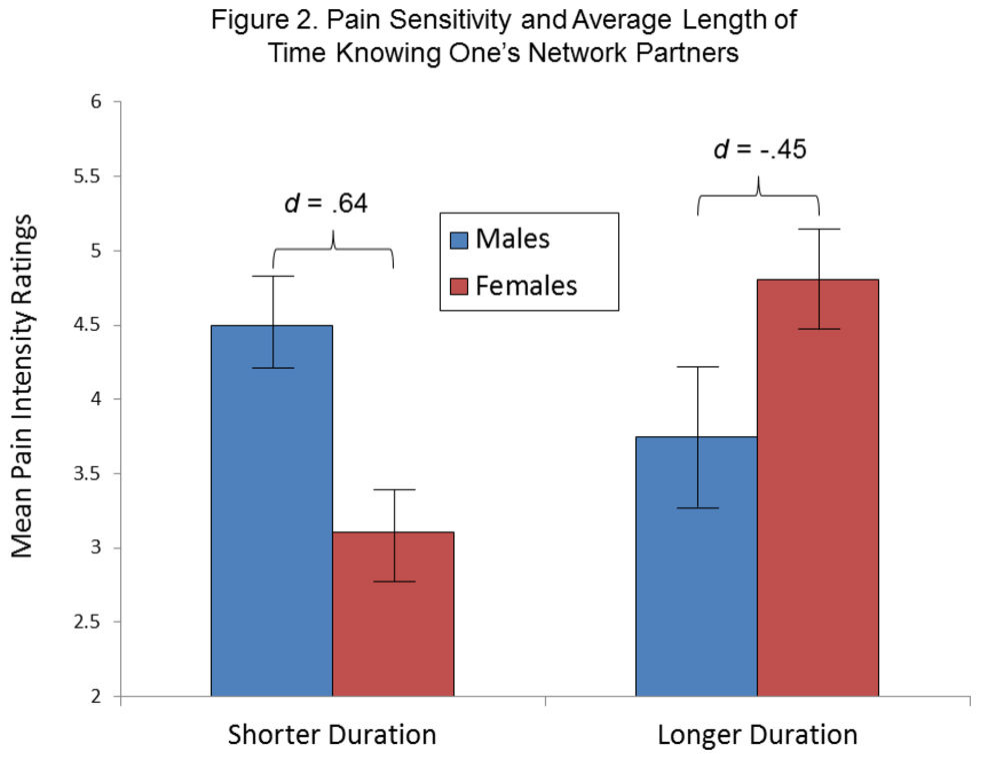
Mean pain intensity ratings according to Sex and Length of Relationships. Length of relationships is represented by values lower than and greater than the sample mean. Bars indicate standard errors of the mean.

 As shown in Table 4, the analysis examining the role of mean length of participants’ relationships revealed a trend for a significant Length of Relationships x Sex interaction term. As shown in Figure 2, this was due to higher pain intensity reports for females who reported longer established relationship, but not for males. The direction of the sex differences was again dependent on the relative level of the social variable, and the effect sizes for the group differences (in opposite directions) were moderate-to-large. 

**Table 4:** ANCOVA Results for Mean Length of Relationships.

Dependent Variable: Pain Intensity					
Source	Type III Sum of Squares	df	Mean Square	F	Sig.
Corrected Model	221.974^a^	9	24.664	6.024	.000
Intercept	366.440	1	366.440	89.494	.000
Duration of hand Immersion	11.154	1	11.154	2.724	.101
Examiner Gender	1.710	1	1.710	.418	.519
Sex	.041	1	.041	.010	.920
Experimental Condition	98.124	1	98.124	23.964	.000
Length of Relationships	5.118	1	5.118	1.250	.266
Sex * Condition	.001	1	.001	.000	.990
Sex * Length	15.738	1	15.738	3.844	.052
Condition * Length	.667	1	.667	.163	.687
Sex * Condition * Length	.008	1	.008	.002	.964
Error	515.918	126	4.095		
Total	3027.333	136			
Corrected Total	737.892	135			

a R Squared = .301 (Adjusted R Squared = .251)

### Relationships with Significant Others

 The final set of equations focused on the role of listing a S/O (e.g., significant other, current partner, boyfriend/girlfriend) among one's network list, and the role of the (functionality) social support scores pertaining to S/O (emotional and logistic support, and length of relationship) on pain intensity, and if sex moderated these associations. An ANCOVA was first used by entering the availability of a S/O (none = 0, listed = 1), Sex, Experimental Condition and the respective two-way and three-way interaction terms as predictor variables, and hand immersion time and examiner gender as covariates for the pain score. As shown in Table 5, there was a trend for a significant S/O x Experimental Condition interaction term. Separate ANCOVAS entering Availability of a S/O, Sex, and the S/O x Sex interaction terms as predictor variables (and hand immersion time and examiner gender as covariates) for participants in both experimental pain conditions did not reveal a significant interaction term or main effect term for Availability of a S/O in the low pain condition. As shown in Table 6, there was a trend for a significant main effect term for S/O in the high pain condition. 

**Table 5:** ANCOVA Results for the Availability of a Significant Other.

Dependent Variable: Pain Intensity					
Source	Type III Sum of Squares	df	Mean Square	F	Sig.
Corrected Model	218.692^a^	9	24.299	5.931	.000
Intercept	369.309	1	369.309	90.149	.000
Duration of Hand Immersion	9.864	1	9.864	2.408	.123
Examiner Gender	1.753	1	1.753	.428	.514
Sex	.091	1	.091	.022	.882
Experimental Condition	130.228	1	130.228	31.789	.000
Availability of a S/O	1.828	1	1.828	.446	.505
Sex * Condition	.385	1	.385	.094	.760
Sex * S/O	1.791	1	1.791	.437	.510
Condition * S/O	18.144	1	18.144	4.429	.037
Sex * Condition * S/O	7.500	1	7.500	1.831	.178
Error	536.662	131	4.097		
Total	3094.778	141			
Corrected Total	755.354	140			

a R Squared = .290 (Adjusted R Squared = .241)

**Table 6:** ANCOVA Results for Availability of a Significant Other in the High Pain Condition.

Dependent Variable: Pain Intensity					
Source	Type III Sum of Squares	df	Mean Square	F	Sig.
Corrected Model	23.144^a^	5	4.629	1.411	.248
Intercept	56.390	1	56.390	17.189	.000
Duration of Hand Immersion	7.790	1	7.790	2.375	.133
Examiner Gender	.385	1	.385	.117	.734
Sex	.000	1	.000	.000	.995
Availability of a S/O	11.135	1	11.135	3.394	.075
Sex * S/O	4.516	1	4.516	1.377	.250
Error	101.697	31	3.281		
Total	1440.889	37			
Corrected Total	124.841	36			

a R Squared = .185 (Adjusted R Squared = .054)

 ANCOVAs were then used to measure the associations between the functionality scores pertaining to S/O (emotional and logistic support, and length of relationship) and pain intensity, and whether sex moderated these associations for the participants who listed a S/O (*n* = 69). The scores (dichotomously coded as lower than and higher than the sample mean values) were entered into three separate ANCOVAs as predictor variables, along with Sex, Experimental Condition, and their respective two-way and three-way interaction terms; hand immersion time and examiner gender were again entered as covariates. None of the interaction terms or main effects pertaining to the emotional support score was significant (*p*s > .10). 

As shown in Table 7, the analysis examining the role of the length of the participant’s relationship with their S/O revealed a trend for a significant Length of Relationship x Experimental Condition interaction term. Separate ANCOVAS entering Length of Relationship with S/O, Sex, and the Length x Sex interaction terms as predictor variables (and hand immersion time and examiner gender as covariates) for participants in both the low pain and high pain conditions did not reveal a significant interaction term or main effect for Length of Relationship in the low pain condition. As shown in Table 8, there was a trend for a significant main effect term for Length of Relationship in the high pain condition. An independent samples *t*-test among participants in the high pain condition showed that participants (males and females combined) who reported having a longer established relationship with their S/O reported lower pain intensity (*M* = 4.92, *SD* = 1.57) than participants with shorter established relationships with their S/O (*M* = 5.93, *SD* = 1.68, *d* = .62). 

**Table 7:** ANCOVA Results for the Length of Relationship with a Significant Other.

Dependent Variable: Pain Intensity					
Source	Type III Sum of Squares	df	Mean Square	F	Sig.
Corrected Model	60.780^a^	9	6.753	1.814	.086
Intercept	142.232	1	142.232	38.197	.000
Duration of Hand Immersion	7.327	1	7.327	1.968	.166
Examiner Gender	5.127	1	5.127	1.377	.246
Sex	.608	1	.608	.163	.688
Experimental Condition	18.892	1	18.892	5.073	.028
Length of Relationship with S/O	.978	1	.978	.263	.610
Sex * Condition	1.969	1	1.969	.529	.470
Sex * Length	.004	1	.004	.001	.974
Condition * Length	13.884	1	13.884	3.729	.059
Sex * Condition * Length	1.404	1	1.404	.377	.542
Error	204.799	55	3.724		
Total	1426.222	65			
Corrected Total	265.579	64			

a R Squared = .229 (Adjusted R Squared = .103)

**Table 8:** ANCOVA Results for the Length of Relationship with a Significant Other in the High Pain Condition.

Dependent Variable: Pain Intensity	
Source	Type III Sum of Squares	df	Mean Square	F	Sig.
Corrected Model	24.921^a^	5	4.984	2.843	.069
Intercept	44.114	1	44.114	25.159	.000
Duration of Hand immersion	9.560	1	9.560	5.452	.040
Examiner Gender	8.711	1	8.711	4.968	.048
Sex	1.583	1	1.583	.903	.362
Length of Relationship with S/O	14.470	1	14.470	8.252	.015
Sex * Length	.642	1	.642	.366	.557
Error	19.288	11	1.753		
Total	549.333	17			
Corrected Total	44.209	16			

a R Squared = .564 (Adjusted R Squared = .365)

**Figure 3:**
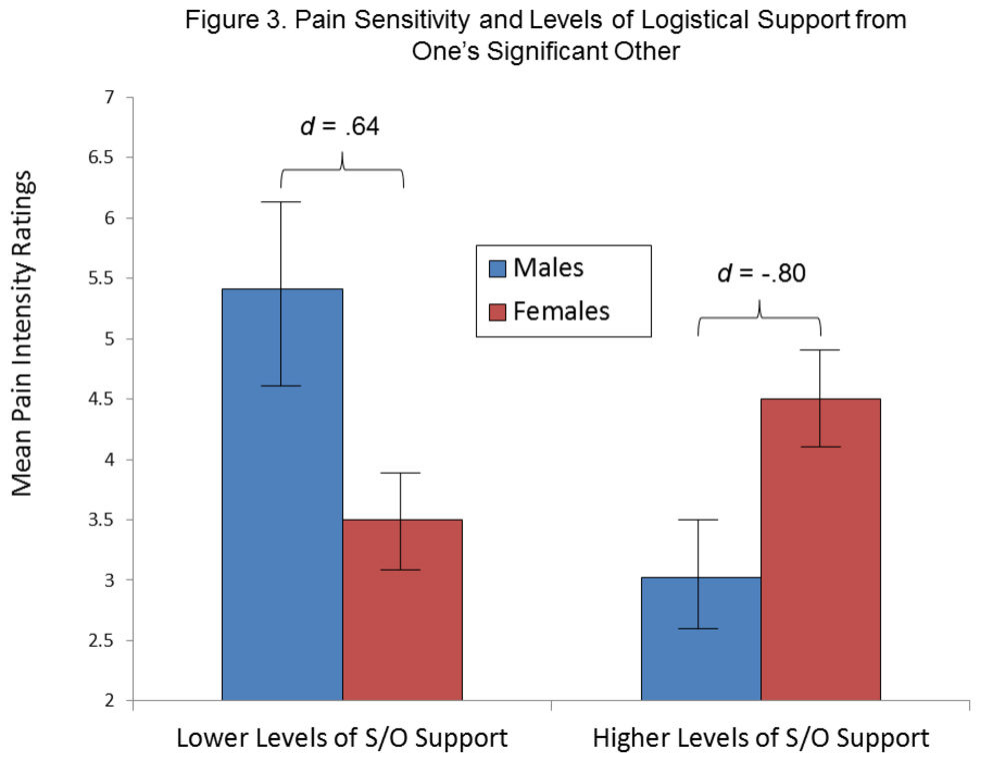
Mean pain intensity ratings according to Sex and Logistical Support received from significant other (S/O). Level of S/O support is represented by values lower than and greater than the sample mean. Bars indicate standard errors of the mean.

As shown in Table 9, the final set of analyses examining the role of logistical support received from one’s S/O revealed a significant Sex x Logistical Support interaction term. Separate ANCOVAS entering Logistical Support from S/O, Experimental Condition, and the Logistical Support x Condition interaction terms as predictor variables (and hand immersion time and examiner gender as covariates) for males and females are shown in Table 10; these analyses revealed a significant main effect term for Logistical Support for males only. Figure 3 shows that the interaction was due to lower pain scores for males who reported higher levels of logistical support and to higher pain scores for females who reported higher levels of logistical support. Figure 3 likewise shows that the nature of the direction of the sex differences in pain intensity was dependent on the social variable, and magnitudes of the effect sizes of the differences (in opposite directions) were large.

**Table 9:** ANCOVA Results for Logistical Support Received from a Significant Other.

Dependent Variable: Pain Intensity					
Source	Type III Sum of Squares	df	Mean Square	F	Sig.
Corrected Model	100.059^a^	9	11.118	3.501	.002
Intercept	109.040	1	109.040	34.334	.000
Duration of Hand Immersion	3.302	1	3.302	1.040	.313
Examiner Gender	.911	1	.911	.287	.594
Sex	2.028	1	2.028	.638	.428
Experimental Condition	17.694	1	17.694	5.571	.022
Logistical Support from S/O	4.600	1	4.600	1.449	.234
Sex * Condition	1.181	1	1.181	.372	.545
Sex * Logistical Support	37.392	1	37.392	11.774	.001
Condition * Logistical Support	.096	1	.096	.030	.863
Sex * Condition * Logistical Support	.273	1	.273	.086	.770
Error	161.970	51	3.176		
Total	1358.889	61			
Corrected Total	262.029	60			

a R Squared = .382 (Adjusted R Squared = .273)

**Table 10:** ANCOVA Results for Logistical Support Received from a Significant Other for Males and Females.

Dependent Variable: Pain Intensity						
Sex	Source	Type III Sum of Squares	df	Mean Square	F	Sig.
Males	Corrected Model	75.778^a^	5	15.156	4.254	.008
	Intercept	55.905	1	55.905	15.691	.001
	Duration of Hand Immersion	.058	1	.058	.016	.900
	Examiner Gender	1.197	1	1.197	.336	.568
	Experimental Condition	17.804	1	17.804	4.997	.036
	Logistical Support from S/O	36.532	1	36.532	10.253	.004
	Condition * Logistical Support	.072	1	.072	.020	.888
	Error	74.823	21	3.563		
	Total	646.111	27			
	Corrected Total	150.601	26			
Females	Corrected Model	35.200^b^	5	7.040	2.589	.048
	Intercept	58.753	1	58.753	21.607	.000
	Duration of Hand Immersion	14.121	1	14.121	5.193	.031
	Examiner Gender	2.159	1	2.159	.794	.380
	Experimental Condition	.988	1	.988	.363	.551
	Logistical Support from S/O	10.401	1	10.401	3.825	.061
	Condition * Logistical Support	.000	1	.000	.000	.990
	Error	76.137	28	2.719		
	Total	712.778	34			
	Corrected Total	111.337	33			

a R Squared = .503 (Adjusted R Squared = .385)

b. R Squared = .316 (Adjusted R Squared = .194)

## Discussion

 This study extends prior findings of the impact of biological sex, social context, and available social support on experimental and clinical pain experiences [1-4,7-22,30-32] by showing that sex differences in one type of exogenous pain percept, cold pressor discomfort intensity, may be dependent on structural and functional dimensions of the individual’s naturalistic peer network. As has been show with previous research, people’s total number of network partners was inversely related to the proportion of intimate affiliates and mean level of logistical support received from their partners [38]; however, none of the social network characteristics (mean levels and levels pertaining to one’s S/O) differed between males and females. Likewise, comparing males and females directly revealed no group differences in pain intensity. However, when individuals’ social networks were considered, inverse sex differences emerged, hence otherwise obscuring the dynamic relations between biological sex and pain performance. Females with a greater proportion of intimate types of relationship partners and more extensive (i.e., longer established) relationships reported higher pain intensity than males, whereas males with less intimate and less established relationships actually reported *higher* pain intensity scores than females, on average. The most robust sex differences were moderated by the amount of logistical support received from one’s significant other such that greater logistical support was associated with dampened pain intensity ratings in males, but with heightened pain intensity in females. Thus, while previous research has found that supportive (e.g., functional) components of social networks are associated with health-related outcomes [9,39,40], this is the first study to show that structural dimensions of social networks are also associated with distinct and potentially adaptive (i.e., epigenetically specialized) expression of pain sensitivity, and that the associations between the social constructs and pain intensity differs for healthy males and females. 

These findings can be interpreted from the social-signaling theory that pain sensations evolved, in part, to heuristically express behaviors (i.e., nonverbal gestures and verbal reports) that demand attention from others, and that the behaviors may ultimately be used to regulate—i.e., attract and deter—interactions with different types of relationship partners [5,6,8,20,21,41]. Pain behaviors may demonstrate vulnerability and by proxy, trustworthiness cues to others, which are similar to other internalizing symptoms (e.g., low mood, worrying) and generally effective at provoking empathetic and solicitous responses from intimate types of relationship partners [5-8]. This thesis leads to the general hypothesis that healthy people who spend a greater amount of time interacting with family members and other intimate partners will experience heightened pain sensitivity. Indeed, numerous studies have shown that higher levels of pain-related social support and solicitous behaviors from significant relationship partners are associated with greater clinical pain experiences [42-50]. Fewer studies have focused on how the individual’s subjective impressions of available social support may influence experimental pain (e.g., CPT) performance [18], and this is the first study to show sex differences in the associations between structural and functional components of social support and pain reports. The current findings are therefore consistent with research showing that people express heightened pain behaviors (e.g., intensity reports and facial expressions) in the presence of intimate affiliates such as a significant other or parents during standard medical procedures [51,52], and the mere presence of a same-sex friend, a female researcher, and female strangers in the immediate context increases experimental pain sensitivity, particularly among women [7,30,32,53]. 

Evolutionary psychology theories of sex differences in expressive styles, including sex differences in the expression of pain behaviors [5,6,8] have attributed the differences to the unique sub-ecologies in which ancestral males and females evolved. According to the social-signaling perspective of pain and more general SRFB [5,6,8,26], these ecologies can be understood from an evolutionary history of *male-male coalitional competition* and *male-biased philopatry* (also referred to as patrilocality or female exogamy). In this type of social system, males tended to remain in closer proximity to their male kin, thus allowing them to form secure, kin-based coalitions, while females tended to emigrate into the social networks of their husbands upon marriage [54]. Greater reliance on non-kin and more distantly-related relationship partners (particularly upon adolescence) would have constrained females to develop higher cognitive thresholds for trusting peers as well as heightened motivations for forming fewer, more time-invested and intimate peer relationships which is necessary for increasing the reliability of social bonds in the absence of inclusive fitness (i.e., shared genes [5,26,54-56]). In theory, these inclinations would have co-evolved with the general behavioral heuristics for females to demonstrate higher levels of altruistic tendencies and vulnerability displays (i.e., non-threat), and hence trustworthiness cues than males, allowing females to strengthen the reliability and security of their peer relationships [5,26,27]. This omnibus thesis helps explain the conventional pattern of women reporting quantitatively and qualitatively (e.g., tied to menstrual functioning) disparate pain experiences, including higher experimental and clinical pain sensitivity, than men on average [1-4,7,8,22,57]. 

A careful extension of this thesis was used to predict the current findings that structural and functional dimensions of individuals’ naturalistic social networks would moderate the relation between biological sex and experimental pain performance. Specifically, the SRFB and social signaling perspective of pain behaviors [5-8] predict that females whose social networks are comprised of a higher proportion of, and more extensive relationships with intimate relationship partners would drive the typical finding of higher pain sensitivity in women. Males were instead predicted to show an inverse association between relationship intimacy and pain intensity due to the hypothesis that males are more likely to utilize empowerment (e.g., prowess and status cues) rather than vulnerability displays to regulate their peer relationships [5,8,26,27]. Moreover, we showed for the first time that the direction of sex differences in experimental pain sensitivity is dependent on the structure and functioning of the individual’s social network. Hence, previous research on sex differences in pain functioning (e.g., neurocognitive and behavioral processes) that did not include concurrent measurements of individuals’ relationship experiences may have missed corollary components of the nexus between biological sex and pain perception. The current findings are also important for highlighting potential within-sex correlates of social psychological functioning, such as gender identity [57-59], which may partly influence external pain perception irrespective of biological sex. Finally, the findings may be especially relevant for understanding sex differences in felt pain caused by extrinsic painful stimuli (e.g., surface, skin tissue damage). It remains unclear at this time how social psychological experiences and additional factors such as affective functioning (e.g., comorbid depression) may contribute to potential tradeoffs in the ability to experience exogenous (e.g., experimental) versus endogenous (e.g., deep tissue, somatic; clinical; chronic) pain sensations (Vigil, unpublished manuscript). 

 In addition to the project’s potentially innovative findings, discussion of limitations is warranted. General methodological limitations are that: a) the study did not control for handedness, which is known to influence CPT measurements [60]; b) reactions to CPT might not predict reactions to other forms of pain; c) results from American university students might not generalize to different ages, cultures, and social network structures; d) self-reports of social network structures and functionality might be biased and noisy; and e) sex differences may be influenced by cultural norms regarding pain sensitivity. It is also likely that initial floor effects confound laboratory discomfort tasks in which felt pain graduates from being nonexistent to being unbearable. Another limitation is that the current study did not measure pain-specific solicitous expectations of the respondents’ relationship partners, thus disallowing a more critical examination of potential psychological mechanisms that may contribute to perceptual processing of pain sensations. Likewise, although we tried to control the potential influence of observer effects, it is still possible that people responded to the virtual presence of the (remote) experimenter in ways that confounded the ability to examine our proposed hypotheses. Finally, since the study is cross-sectional, the presumed influence of social experiences on pain sensitivity can only be considered tentative, and it is possible that the constructs have bi-directional relationships. It is plausible, for instance, that males with higher pain sensitivity (e.g., ‘wimpy guys’) have fewer friends, and that socially expressive females tend to have more friends. These alternative possibilities are also consistent with the social-signaling perspective of pain functioning and the general thesis that males and females utilize pain behaviors in somewhat specialized and selective ways (e.g., pain concealment vs. pain reactivity) for regulating their peer relationships [5-8,26,27].

 In conclusion, the current findings contribute to previous research on the role of biological sex and social support on pain behaviors by showing that the associations between pain reporting and structural and functional components of individuals’ social networks are dynamically and uniquely expressed in males and females. Although it is not axiomatic that the findings would transfer to clinical settings, the observed patterns may have indirect implications for managing patient care, including understanding how patients’ social networks may influence their pain experiences and overall medical prognosis and for developing more individualized pain treatment options (e.g., relationship counseling) for patients that may be receptive to such interventions. Nonetheless, the current findings are consistent with the thesis that there may be evolved cost-benefit fitness tradeoffs associated with forming and maintaining different types of relationships, and these tradeoffs may partly modulate specialized and functional and hence adaptive pain sensitivities in healthy males and females. 
